# Supplementary material for: Identification and analysis of the expansin gene family in yam
Source: PeerJ. 2025 Sep 30;13:e20093. doi: 10.7717/peerj.20093 (PMC12493719; doi:10.7717/peerj.20093)

**MIQE checklist**

| **ITEM TO CHECK** | **IMPORTANCE** | **CHECKLIST** |
| --- | --- | --- |
| **EXPERIMENTAL DESIGN** |  |  |
| Definition of experimental and control groups | **E** | The expression levels of each gene during the NH-F stage serving as the control group, and other stages as the experimental group for analysis. |
| Number within each group | **E** | on page 7, line 223-225 |
| Assay carried out by core lab or investigator's lab? | **D** | investigator's lab |
| Acknowledgement of authors' contributions | **D** | Supplementary Material |
| **SAMPLE** |  |  |
| Description | **E** | on page 7, line 217-218 |
| Volume/mass of sample processed | **D** | NA(Not Applicable) |
| Microdissection or macrodissection | **E** | on page 7, line 223-224 |
| Processing procedure | **E** | on page 7, line 223-226 |
| If frozen - how and how quickly? | **E** | on page 7, line 225-226 |
| If fixed - with what, how quickly? | **E** | NA |
| Sample storage conditions and duration (especially for FFPE samples) | **E** | on page 7, line 225-226 |
| **NUCLEIC ACID EXTRACTION** |  |  |
| Procedure and/or instrumentation | **E** | on page 7, line 228-229 |
| Name of kit and details of any modifications | **E** | on page 7, line 228-229 |
| Source of additional reagents used | **D** | NA |
| Details of DNase or RNAse treatment | **E** | RNase was not available in the operating environment, reagents and consumables. |
| Contamination assessment (DNA or RNA) | **E** | on page 7, line 229-231 |
| Nucleic acid quantification | **E** | on page 7, line 230-231 |
| Instrument and method | **E** | on page 7, line 229-231 |
| Purity (A260/A280) | **D** | greater than 1.80(See after the table) |
| Yield | **D** | NA |
| RNA integrity method/instrument | **E** | on page 7, line 229-231 |
| RIN/RQI or Cq of 3' and 5' transcripts | **E** | Supplementary Material |
| Electrophoresis traces | **D** | See after the table |
| Inhibition testing (Cq dilutions, spike or other) | **E** | NA |
| **REVERSE TRANSCRIPTION** |  |  |
| Complete reaction conditions | **E** | NA |
| Amount of RNA and reaction volume | **E** | 1 µg total RNA and 20 µL reaction volume |
| Priming oligonucleotide (if using GSP) and concentration | **E** | NA |
| Reverse transcriptase and concentration | **E** | M-MLV reverse transcriptase |
| Temperature and time | **E** | 50°C for 15 minutes and 85°C for 5 seconds. |
| Manufacturer of reagents and catalogue numbers | **D** | on page 7, line 231-233 |
| Cqs with and without RT | **D*** | Not determined |
| Storage conditions of cDNA | **D** | cDNA was stored at −20 °C |
| **qPCR TARGET INFORMATION** |  |  |
| efficiency and LOD of each assay. | **E** | NA |
| Sequence accession number | **E** | on page 7, line 233 |
| Location of amplicon | **D** | NA |
| Amplicon length | **E** | See after the table |
| *In silico* specificity screen (BLAST, etc) | **E** | NCBI Primer-BLAST |
| Pseudogenes, retropseudogenes or other homologs? | **D** | **no** |
| Sequence alignment | **D** | NA |
| Secondary structure analysis of amplicon | **D** | NA |
| Location of each primer by exon or intron (if applicable) | **E** | NA |
| What splice variants are targeted? | **E** | NA |
| **qPCR OLIGONUCLEOTIDES** |  |  |
| Primer sequences | **E** | Supplementary Material  (Table S5) |
| RTPrimerDB Identification Number | **D** | NA |
| Probe sequences | **D**** | NA |
| Location and identity of any modifications | **E** | NA |
| Manufacturer of oligonucleotides | **D** | Sangon Biotech (Shanghai) |
| Purification method | **D** | NA |
| **qPCR PROTOCOL** |  |  |
| Complete reaction conditions | **E** | Each qPCR reaction was performed in a 10 µL volume containing 1.0 µL of cDNA, 0.4 µL each of forward and reverse primers (10 µM final concentration), 3.2 µL of nuclease-free water, and 5.0 µL of AceQ qPCR SYBR Green Master Mix (Vazyme, Nanjing, China) |
| Reaction volume and amount of cDNA/DNA | **E** | 10 µL volume containing 1.0 µL of cDNA, |
| Primer, (probe), Mg^2+^ and dNTP concentrations | **E** | forward and reverse primers (10 µM final concentration) |
| Polymerase identity and concentration | **E** | on page 7, line234-235 |
| Buffer/kit identity and manufacturer | **E** | on page 7, line234-235 |
| Exact chemical constitution of the buffer | **D** | NA |
| Additives (SYBR Green I, DMSO, etc.) | **E** | SYBR Green I |
| Manufacturer of plates/tubes and catalog number | **D** | 0.1 ml No-Skirted 96-Well PCR Plates(Vazyme, Nanjing, China) |
| Complete thermocycling parameters | **E** | Real-Time System with the following program: 95°C for 3 minutes , followed by 39 cycles of 95°C for 10 seconds and 55°C for 30 seconds with plate reading. A melt curve analysis was performed from 65°C to 95°C with a 0.5°C increment and a 5 second hold at each step. |
| Reaction setup (manual/robotic) | **D** | manual setting |
| Manufacturer of qPCR instrument | **E** | on page 7, line235-236 |
| **qPCR VALIDATION** |  |  |
| Evidence of optimisation (from gradients) | **D** | NA |
| Specificity (gel, sequence, melt, or digest) | **E** | Single peaks in the melt curves confirmed primer specificity. |
| For SYBR Green I, Cq of the NTC | **E** | greater than 35 |
| Standard curves with slope and y-intercept | **E** | NA |
| PCR efficiency calculated from slope | **E** | NA |
| Confidence interval for PCR efficiency or standard error | **D** |  |
| r2 of standard curve | **E** | NA |
| Linear dynamic range | **E** | NA |
| Cq variation at lower limit | **E** | NA |
| Confidence intervals throughout range | **D** |  |
| Evidence for limit of detection | **E** | NA |
| If multiplex, efficiency and LOD of each assay. | **E** | NA |
| **DATA ANALYSIS** |  |  |
| qPCR analysis program (source, version) | **E** | SPSS Statistics (version 22.0, IBM Corporation) |
| Cq method determination | **E** | using a fixed fluorescence threshold. |
| Outlier identification and disposition | **E** | Cq values deviating more than three standard deviations from the mean, Such samples were retested |
| Results of NTCs | **E** | Cq values either undetectable or greater than 35 |
| Justification of number and choice of reference genes | **E** | The actin gene *DoActin* (GenBank accession no. KU669295) as the reference gene. |
| Description of normalisation method | **E** | The comparative Ct method  NH-F stage serving as the control group, and other stages as the experimental group for analysis. |
| Number and concordance of biological replicates | **D** | three independent biological replicates |
| Number and stage (RT or qPCR) of technical replicates | **E** | three technical replicates |
| Repeatability (intra-assay variation) | **E** | NA |
| Reproducibility (inter-assay variation, %CV) | **D** | NA |
| Power analysis | **D** | NA |
| Statistical methods for result significance | **E** | The data were analysed with one-way ANOVA with P<0.05 indicating significant differences. |
| Software (source, version) | **E** | SPSS Statistics (version 22.0, IBM Corporation)  GraphPad Prism 8.0. |
| Cq or raw data submission using RDML | **D** | see Supplementary File 11 --The Cq values for qPCR of DoEXP genes |

Electrophoresis traces：


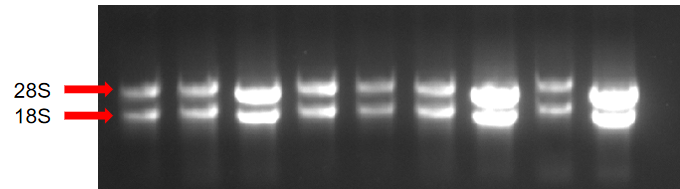
Note：Partial representative results

Purity (A260/A280) and Yield

| Period | Sample batch | Nucleic acid detection | ng/μl | A260/A280 | A260/A230 |
| --- | --- | --- | --- | --- | --- |
| NH_F | 1 | RNA | 260.6 | 2.07 | 1.95 |
| NH_E | 1 | RNA | 368.1 | 2.11 | 2.19 |
| NH_M | 1 | RNA | 187.3 | 2.08 | 2.06 |
| NH_L | 1 | RNA | 202.9 | 2.09 | 2.15 |
| NH_F | 2 | RNA | 633.7 | 2.12 | 2.16 |
| NH_E | 2 | RNA | 433.1 | 2.15 | 2.25 |
| NH_M | 2 | RNA | 335.8 | 2.12 | 2.07 |
| NH_L | 2 | RNA | 167.9 | 2.09 | 2.05 |
| NH_F | 3 | RNA | 413.6 | 2.17 | 2.14 |
| NH_E | 3 | RNA | 812.7 | 2.15 | 2.27 |
| NH_M | 3 | RNA | 351.3 | 2.15 | 2.19 |
| NH_L | 3 | RNA | 203.4 | 2.14 | 2.15 |

Amplicon length:


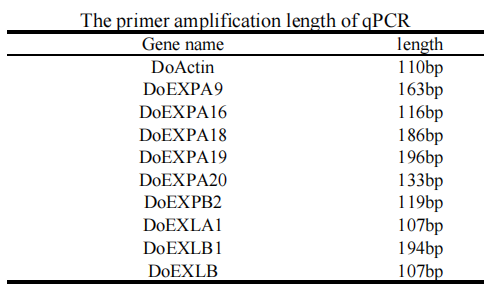

Supplement: Supplemental Information 1 [file peerj-13-20093-s001.docx]
