# Supplementary material for: Identification and analysis of the expansin gene family in yam
Source: PeerJ. 2025 Sep 30;13:e20093. doi: 10.7717/peerj.20093 (PMC12493719; doi:10.7717/peerj.20093)
Supplement: Supplemental Information 2 [file peerj-13-20093-s002.pdf]

motif	width	sites	Logo	consensus sequence	e-value
1	29	30	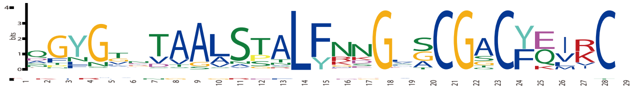	QGYGTNTAALSTALFNNGLSCGACYZIRC	1.5e-408
2	26	20	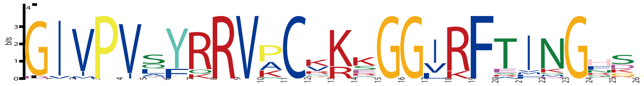	GIVPVSYRRVPCKKKGGIRFTINGHS	4.1e-305
3	37	18	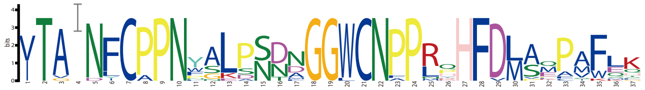	VTATNFCPPNYALPSBNGGWCNPPRQHFDLAQPAFLK	2.0e-429
4	41	22	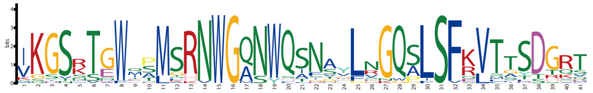	IKGSRTGWMPMSRNWGQNWQSNAYLNGQSLSFKVTTSDGRT	2.1e-471
5	29	24	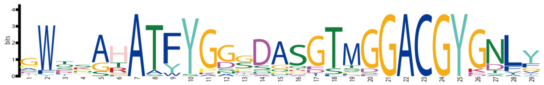	GWQSAHATFYGGGDASGTMGGACGYGNLY	1.5e-329
6	21	30	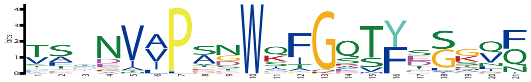	TSYNVAPSNWQFGQTYESGQF	1.4e-193
7	15	23	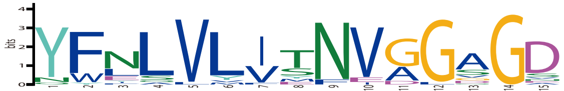	YFNLVLITNVGGAGD	1.1e-152
8	32	9	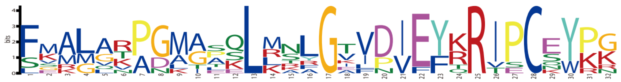	FMALARPGMAPQLMNLGTVDIEYKRIPCEYPG	1.7e-076
9	49	7	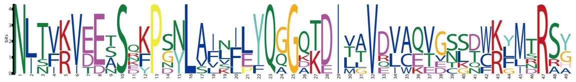	NLTVKVEESSQKPSNLAILILYQGGQTDILAVDVAQVGSSDWKYMTRSY	1.9e-096
10	11	18	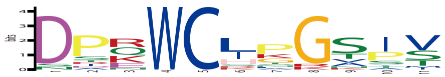	DPRWCLPGSIV	6.7e-054
