# Supplementary material for: Identification and analysis of the expansin gene family in yam
Source: PeerJ. 2025 Sep 30;13:e20093. doi: 10.7717/peerj.20093 (PMC12493719; doi:10.7717/peerj.20093)
Supplement: Supplemental Information 3 — Information on tandem and segmental duplication pairs of DoEXPs, along with the collinearity color for segmental duplication gene pairs. [file peerj-13-20093-s003.pdf]

tandem duplication	segmental duplication	collinearity with colors
DoEXPA14--DoEXPA15	DoEXPA17--DoEXPA2	
DoEXLA1--DoEXLA2	DoEXPA19--DoEXPA2	
	DoEXPA2--DoEXPA4	
	DoEXPA17--DoEXPA4	
	DoEXPA19--DoEXPA4	
	DoEXPA21--DoEXPA5	
	DoEXPA5--DoEXPA7	
	DoEXPA10--DoEXPA6	
	DoEXPA18--DoEXPA6	
	DoEXPA6--DoEXPA8	
	DoEXPA21--DoEXPA7	
	DoEXPA10--DoEXPA8	
	DoEXPA18--DoEXPA8	
	DoEXPA10--DoEXPA18	
	DoEXPA12--DoEXPA20	
	DoEXPA17--DoEXPA19	
