# Supplementary material for: Identification and analysis of the expansin gene family in yam
Source: PeerJ. 2025 Sep 30;13:e20093. doi: 10.7717/peerj.20093 (PMC12493719; doi:10.7717/peerj.20093)
Supplement: Supplemental Information 4 [file peerj-13-20093-s004.pdf]

Arabidopsis thaliana&Dioscorea opposite				
Seq_1	Seq_2	Ka	Ks	Ka/Ks
DoEXPA7	transcript:AT5G05290.1	0.199863	2.166036	0.092271
DoEXPA5	transcript:AT2G40610.1	0.164219	1.829676	0.089753
DoEXPA21	transcript:AT2G40610.1	0.162790	4.129702	0.039419
DoEXPA17	transcript:AT1G26770.2	0.136918	2.421505	0.056543
DoEXPA17	transcript:AT1G69530.3	0.149440	2.378804	0.062822
DoEXPA17	transcript:AT2G03090.1	0.147598	2.319096	0.063645
DoEXPA17	transcript:AT3G29030.1	0.195687	1.649326	0.118647
DoEXPA2	transcript:AT1G26770.2	0.146796	2.909061	0.050462
DoEXPA2	transcript:AT2G03090.1	0.162012	1.949714	0.083095
DoEXPA2	transcript:AT3G29030.1	0.198754	2.321900	0.085600
DoEXPA8	transcript:AT2G37640.1	0.206143	2.013714	0.102370
DoEXPA8	transcript:AT2G28950.1	0.199398	2.169781	0.091898
DoEXPA8	transcript:AT2G39700.1	0.200331	1.620428	0.123628
DoEXPA8	transcript:AT3G55500.1	0.202002	2.419012	0.083506
DoEXPA8	transcript:AT5G02260.1	0.232889	1.575343	0.147834
DoEXPA10	transcript:AT2G37640.1	0.154979	2.794781	0.055453
DoEXPA10	transcript:AT3G55500.1	0.159427	1.900820	0.083873
DoEXPA10	transcript:AT5G02260.1	0.177932	2.252721	0.078985
DoEXPA6	transcript:AT2G39700.1	0.138734	2.490171	0.055712
DoEXPA6	transcript:AT2G37640.1	0.155401	3.036225	0.051182
DoEXPA6	transcript:AT3G55500.1	0.160615	1.736678	0.092484
DoEXPA6	transcript:AT5G02260.1	0.194523	2.000085	0.097257
DoEXPA18	transcript:AT2G37640.1	0.158734	1.905545	0.083301
DoEXPA18	transcript:AT3G55500.1	0.163897	2.688968	0.060952
DoEXPA18	transcript:AT5G02260.1	0.206624	1.766376	0.116976
DoEXPA9	transcript:AT1G20190.1	0.257717	2.345417	0.109881
DoEXPB1	transcript:AT2G45110.1	0.373977	3.101537	0.120578
DoEXPB1	transcript:AT3G60570.1	0.502348	2.378804	0.211177
