# Supplementary material for: Identification and analysis of the expansin gene family in yam
Source: PeerJ. 2025 Sep 30;13:e20093. doi: 10.7717/peerj.20093 (PMC12493719; doi:10.7717/peerj.20093)
Supplement: Supplemental Information 5 [file peerj-13-20093-s005.pdf]

Oryza sativaL&Dioscorea opposite				
Seq_1	Seq_2	Ka	Ks	Ka/Ks
DoEXPA7	transcript:Os01t0823100-01	0.164087	2.463195	0.066616
DoEXPA5	transcript:Os01t0823100-01	0.167376	4.281238	0.039095
DoEXPA8	transcript:Os03t0822000-02	0.175115	1.432076	0.122280
DoEXPA10	transcript:Os03t0822000-02	0.121103	1.074208	0.112737
DoEXPA18	transcript:Os03t0822000-02	0.135886	1.321129	0.102856
DoEXPA13	transcript:Os04t0583500-01	0.209221	1.180361	0.177252
DoEXPA16	transcript:Os06t0621900-01	0.319095	2.089034	0.152748
DoEXPB1	transcript:Os04t0552000-00	0.299438	2.311520	0.129542
DoEXPB2	transcript:Os04t0530100-01	0.451159	2.424565	0.186078
DoEXLB1	transcript:Os07t0496250-01	0.259178	1.528103	0.169608
