# Supplementary material for: Identification and analysis of the expansin gene family in yam
Source: PeerJ. 2025 Sep 30;13:e20093. doi: 10.7717/peerj.20093 (PMC12493719; doi:10.7717/peerj.20093)
Supplement: Supplemental Information 6 [file peerj-13-20093-s006.pdf]

Gene Name	Forward primer(5'-3')	Reverse primer(5'-3')
DoActin	GAGCAAGGAAATCACAGCAC	TCAGGGAAGCCAAGATAGAG
DoEXPA9	CTGCGTGCGGACAATGCTA	AAATGCTGGCGAGGAGGGT
DoEXPA16	GCCACCTACTCCAAAGACA	AACAAAGCACCGCTCACT
DoEXPA18	CACATTCCAGGCGTCTTC	AGCTCCACAGCTCATCCC
DoEXPA19	AGAAAGGCGGAATAAGGT	TTGAAGGACAGTGGCTGA
DoEXPA20	GTGGGTGAAGGGCAGTAA	GATGGTTTGGCGAGTGGT
DoEXPB2	TCTCAGTGGTGCTGCTTT	TGTTCTTTCCTGGATACTTG
DoEXLA1	GGATGGCTCTGAAACTCA	GCTGGTCTCCTCCACTCT
DoEXLB1	GGAGCGAGTGGAAACACC	GCCAAGTAGCCTGGGAAG
DoEXLB2	ACTACTTGGCACTTGTAATCC	ATAAGCCCTCCTCATCCC
