# Supplementary figures and images for: Identification and analysis of the expansin gene family in yam
Source: PeerJ. 2025 Sep 30;13:e20093. doi: 10.7717/peerj.20093 (PMC12493719; doi:10.7717/peerj.20093)

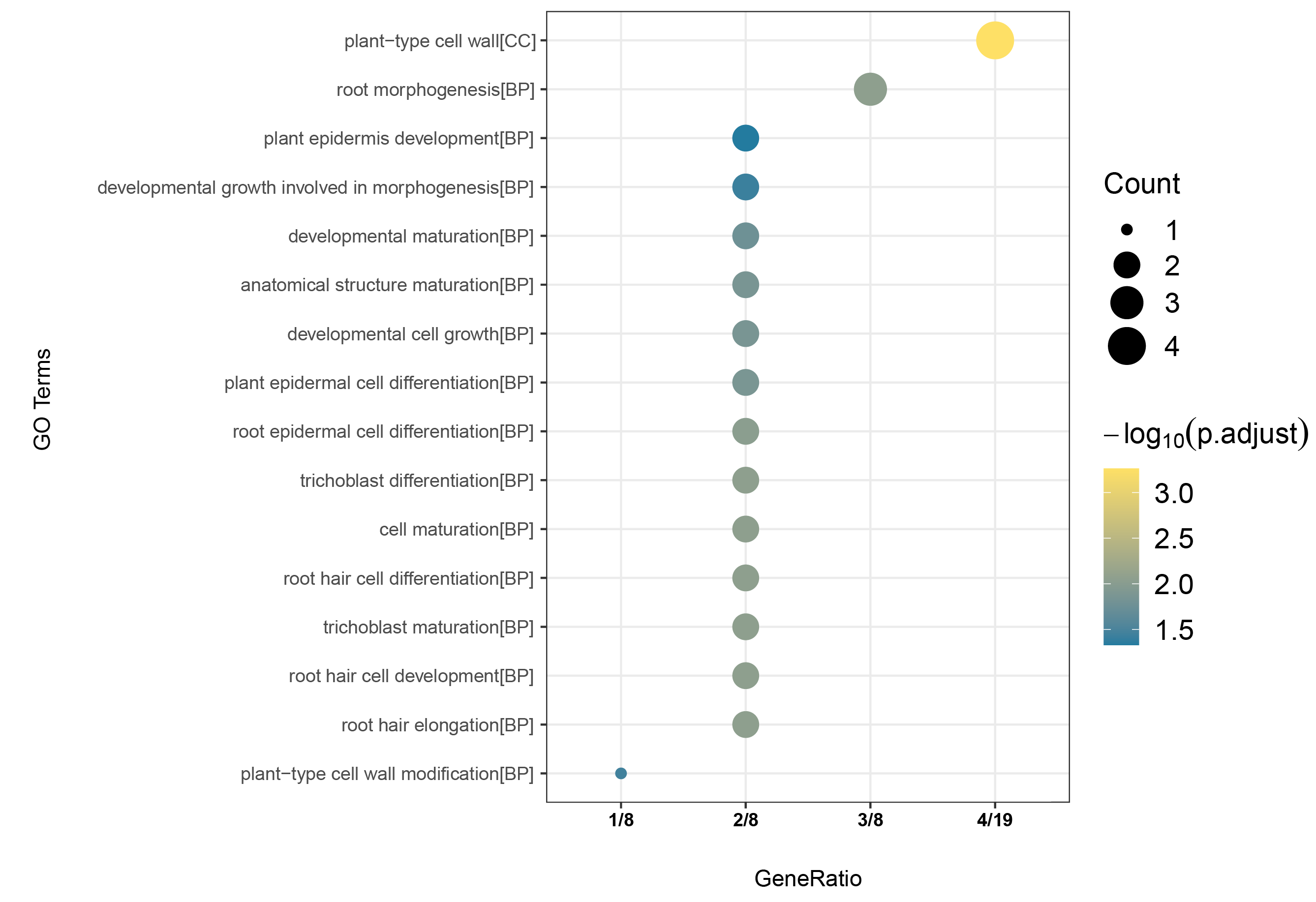

Supplement: Supplemental Information 7 — Vertical axis represents the enriched GO terms, with module information for each term provided in parentheses; Horizontal axis shows the proportion of significantly enriched genes (corrected P-value < 0.05) in DoEXPs relative to the total enriched genes within the corresponding module; Count (represented by circle size) indicates the number of DoEXP genes enriched per GO term; Bubble color reflects corrected P-values, with a gradient from yellow to blue indicating increasing statistical significance (i.e., decreasing P-value). [file peerj-13-20093-s007.png]
