# Supplementary material for: Identification and analysis of the expansin gene family in yam
Source: PeerJ. 2025 Sep 30;13:e20093. doi: 10.7717/peerj.20093 (PMC12493719; doi:10.7717/peerj.20093)
Supplement: Supplemental Information 10 [file peerj-13-20093-s010.docx]

**Abbreviations list**

*DoEXP*s: *Dioscorea opposita* expansin genes

NJ: Neighbor-joining

G2A: Group 2 grass pollen allergen proteins

MEME: Multiple em for motif elicitation

MeJA: Methyl jasmonate

ABA: Abscisic acid

GA: Gibberellin

IAA: Auxin

SA: Salicylic acid

MYB: Myeloblastosis

MBS: Drought-inducible MYB Binding Site

LTR: Low-temperature-responsive

Ka: The nonsynonymous substitution rate

Ks: The synonymous substitution rate

Mya: Million years ago

PFAM: Database of protein families

GO:Gene ontology

CC: Cellular component

BP: Biological process

MF: Molecular function

qPCR: Real-time quantitative polymerase chain reaction
